# Ontology-Defined Middleware for Internet of Things Architectures

**DOI:** 10.3390/s19051163

**Published:** 2019-03-07

**Authors:** Víctor Caballero, Sergi Valbuena, David Vernet, Agustín Zaballos

**Affiliations:** Engineering Department, Universitat Ramon Llull (URL), La Salle, 08022 Barcelona, Spain; sergi.valbuena@students.salle.url.edu (S.V.); david.vernet@salle.url.edu (D.V.); agustin.zaballos@salle.url.edu (A.Z.)

**Keywords:** heterogeneity, middleware, semantic, ontology, behaviour, web-of-things

## Abstract

The Internet of Things scenario is composed of an amalgamation of physical devices. Those physical devices are heterogeneous in their nature both in terms of communication protocols and in data exchange formats. The Web of Things emerged as a homogenization layer that uses well-established web technologies and semantic web technologies to exchange data. Therefore, the Web of Things enables such physical devices to the web, they become Web Things. Given such a massive number of services and processes that the Internet of Things/Web of Things enables, it has become almost mandatory to describe their properties and characteristics. Several web ontologies and description frameworks are devoted to that purpose. Ontologies such as SOSA/SSN or OWL-S describe the Web Things and their procedures to sense or actuate. For example, OWL-S complements SOSA/SSN in describing the procedures used for sensing/actuating. It is, however, not its scope to be specific enough to enable a computer program to interpret and execute the defined flow of control. In this work, it is our goal to investigate how we can model those procedures using web ontologies in a manner that allows us to directly deploy the procedure implementation. A prototype implementation of the results of our research is implemented along with an analysis of several use cases to show the generality of our proposal.

## 1. Introduction

The recent growth of the Internet has fostered the interaction between many heterogeneous technologies. The term Internet of Things (IoT) [1] has been coined as the umbrella term that refers to this new reality. The heterogeneity of the IoT encompasses both hardware and software. At the hardware level, an amalgamation of devices from multiple vendors take part in the IoT. At the software level, those devices use multiple Internet protocols and data exchange formats. Device manufacturers choose what seems to fit their needs in describing and operating their devices. Hence, the current IoT scenario is filled with devices/Things that are not fully interoperable. Even stronger lack of interoperability can be found in vertical domain specific silos of IoT. In order to facilitate the communication between devices and the integration of them into systems and systems of systems, researchers have conducted work that aims at providing a common communication interface. The Web of Things (WoT) [2] provides a common interface by translating the heterogeneity into the homogeneity of the Web 3.0. To achieve a common language, the WoT refers to Hyper-Text Transfer Protocol/Secure (HTTP/S) as the standard communication protocol and to Semantic Web [3] as the common information exchange structure.

An example that takes advantage of both IoT and WoT technologies is as follows. In the context of a Smart Home, there are several electrical appliances which consume or produce energy. On the one hand, home appliances that consume energy can be an air conditioner, vacuum cleaner, washing machine, clothes dryer, dishwasher, oven, stove, mobile charger, lights and so on. On the other hand, devices that produce energy usually take advantage of Renewable Energy Sources (RESs) like photo-voltaic panels. In order to take advantage of both production and consumption, those devices need to exchange information between themselves. A course of action would be, for example, to autonomously turn on the clothes dryer during the day when there is enough sunlight to produce enough energy for it to function. It is very unlikely, though, that they all use the same communication protocol and data exchange format. Some will use wireless protocols like Bluetooth, ZigBee, Z-Wave or 6LoWPAN, or wired ones like Ethernet, RS-232, or USB, to name a few. The WoT integration pattern proposes to use gateways as translators between those protocols (some are proprietary) and HTTP/S, along with data exchange format translation, which occurs from a variety of formats such as EXI, JSONB or unstructured data to a semantically annotated Web 3.0 standards, such as JSON or XML. Scenarios like the one presented are very likely to occur. Each scenario, however, will comprehend different types of devices that use different protocols and different data exchange formats. The simple scenario of pairing a light bulb from brand A with a switch from brand B can be different in each household. In such a simple use case, the task of describing each specific scenario can be tedious. Therefore, there is a need to describe the execution flow or behaviour of those devices in an abstract manner, thus the description can be used for multiple and diverse devices with the same capabilities.

### 1.1. Motivation

A large number of heterogeneous devices share the same capabilities and concrete services, with concrete, shared behaviours can be requested from them. Normally, the description of the services and the execution process that they follow are specified and implemented in multiple manners, which pose an obstacle for the shareability and interpretation of their execution flow. A great effort in providing a common abstraction and interoperability layer is being done in the framework of the IoT/WoT. The World Wide Web Consortium (W3C), for example, is leading an effort in order to provide a common communication and abstraction layer. There is also a growing tendency to describe services using Semantic Web standards such as OWL-S [4], W3C Web of Things (WoT) Thing Description (TD) [5] or the oneM2M Base Ontology [6]. Our vision is that we can share and reuse a concrete flow of execution among multiple and heterogeneous IoT deployments using existing standards. We can leverage Semantic Web Ontologies to model, and execute by means of a specialized engine, the behaviour of the devices that lay in the lower layer of the IoT, by providing a common template (abstraction) that defines such behaviour. This template can be reused by as many Things and services as needed in a variety of heterogeneous deployments.

### 1.2. Contributions

This work aims to address the challenge of describing and executing the same behaviour in multiple and heterogeneous IoT scenarios. In heterogeneous IoT scenarios, where devices from different manufacturers are aimed at providing the same service, it is often found that their heterogeneity prevents developers from reusing the same solution for each device. We address that challenge by using Finite-State Machines (FSMs) to model their behaviour, translating the FSM into in abstract description framework, namely Resource Description Framework (RDF) [7], and developing an engine capable of interpreting and executing the RDF model. The main contributions of this work are:

1. Abstraction of flow of control or behaviour over the heterogeneity of the IoT using existing standards such as RDF, SPARQL Protocol and RDF Query Language (SPARQL), FSM and HTTP/S. This abstraction brings reusability.
2. A prototype implementation of an engine and web interface that allows direct deployment, interpretation and execution of a concrete instance obtained by materializing the abstraction.
3. Analysis of several use cases that demonstrates the generality of the abstraction.

This article is organized as follows. Section 2 gives a brief overview of IoT/WoT standards relevant to our goal and analyses related work on control of flow/behaviour modelling using Semantic Web technologies. Section 3 gives an overview of the architecture that supports our approach. Section 4 introduces and models the use case that is used to explain our solution and Section 5 enhances the model using ontologies. Section 6 explains how we use the template (abstraction) and execute the behaviour described in it. Section 7 analyses several use cases in relation to our approach and finally Section 8 concludes the paper and presents future work.

## 2. State of the Art

Given the massive number of services and processes that IoT enables, it has become almost mandatory to describe such services and processes to enhance interoperability, allowing them to be automatically discovered and called by multiple, heterogeneous systems [8]. Ontologies such as OWL-S [4] and low-level specifications such as the TD [5] or the oneM2M Base Ontology [6] can be used together to describe IoT/WoT systems, fostering interoperability. OWL-S helps software agents to discover the web service that will fulfil a specific need. Once discovered, OWL-S provides the necessary language constructs to describe how to invoke the service. It allows describing inputs and outputs. Thanks to the high-level description of the service, it is possible to compose multiple services to perform more complex tasks. In OWL-S, service description is organized into four areas: the *process model*, the *profile*, the *grounding* and the *service*. Specifically, the process model describes how a service performs its tasks. It includes information about inputs, outputs, preconditions and results. Similarly, the oneM2M Global Initiative [9] defines a standard for machine-to-machine communication interoperability at the semantic level, the one M2M Base Ontology, which is a high-level ontology designed to foster interoperability among other ontologies using equivalences and alignments. The TD “is a central building block in the W3C Web of Things (WoT) and can be considered as the entry point of a Thing [...]. The TD consists of semantic metadata for the Thing itself, an interaction model based on WoT’s Properties, Actions, and Events paradigm, a semantic schema to make data models machine-understandable, and features for Web Linking to express relations among Things”. Both oneM2M Base Ontology and the ontology defined in TD strive for interoperability among multiple IoT applications and platforms, each one covering a large set of use cases, so there is also a work in progress to align the oneM2M ontology with the TD ontology [10]. Orthogonal to these ontologies, the SOSA/SSN ontology is an ontology for describing sensors and their observations. Among other concepts, it defines the class sosa:Procedure, which “is a reusable workflow, protocol, plan, algorithm, or computational method that can be used, among others, to specify how an observation activity has been made [...], (the sosa:Procedure) can be either a record of how the actuation has been performed or a description of how to interact with an actuator (i.e., the recipe for performing actuations)” [11]. How much detail is provided to define such procedures is beyond the scope of SOSA/SSN. It is our vision that detailed and deployable procedure descriptions could fit into and be orthogonal to the models just presented. This work aims at modelling the flow of control for a given service. Therefore, our focus is on modelling the procedure/behaviour of Things using ontologies in a manner that allows us to directly deploy the behaviour implementation.

Work aimed at modelling the behaviour of Things using FSMs and Web Ontology Language (OWL) exists in the literature. On [12], they aim to represent Unified Modeling Language (UML) FSMs using OWL, performing an almost one-to-one translation between UML concepts and OWL classes. Although their mapping from UML to OWL allows for a more machine-readable information structure, its complexity makes it unpractical to use for our objective. The work done in [13] presents a simpler FSM model. UML is used to specify platform independent navigation guides in web applications. They use OWL to describe a model for FSMs which serves as a meta-model for semantic web descriptions of the navigation guides on the Semantic Web. There also exists some scientific literature devoted not only to create a model to express the behaviour of a service but also to interpret and execute the behaviour. In [14], the aim is to develop an FSM that a special server can read and translate to executable entities. These executable entities are executed later by robots. They successfully satisfy their objective, their OWL FSM is domain specific and it includes properties that solve the complexity of their use case but make the FSM too complex for our goal. The goal in [15] is to model an FSM that can be easily translated into Programmable Logic Controller (PLC) code. This model is domain-agnostic as it is not directly merged with the PLC domain. Works [14,15] show how a semantic FSM can be translated and executed by machines, although their application field is industrial and not on the WoT. In our approach, we do not want to express or translate the model in languages other than OWL so it maintains the benefits that OWL gives. Our aim is to build a model that is directly interpreted by different machines.

Our solution aims to be as middleware-agnostic as possible, being a building block for middleware that implement other technical challenges. Therefore, we rely on standard and well-known architectures to contextualize our proposal. We have considered two big standardization and recommendation bodies devoted to promoting interoperability among IoT verticals. An international partnership project for IoT standards, the oneM2M Global Initiative [9] aims to minimize M2M service layer standards by consolidating standardization activities among several member organizations. The W3C Web of Things Working Group [5] aims to develop standards for the Web of Things; their goal is also to reduce fragmentation between IoT silos. For this work, we are interested in the architecture standards and recommendations that oneM2M and W3C WoT Working Group propose. As our objective is to contextualize and provide a prototype implementation for our solution, the W3C WoT Architecture recommendation seems more suitable for that purpose for its simplicity. The architecture is explained in Section 3.

## 3. Architecture

**Figure 1:**
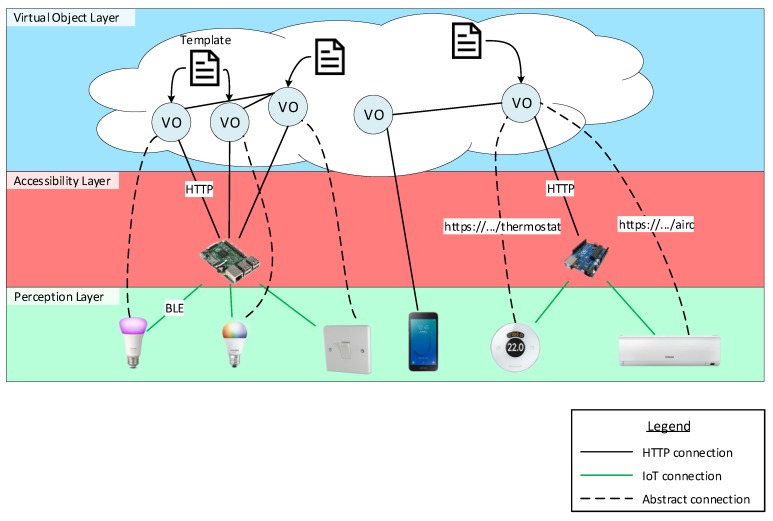
Network architecture used in our proposal. Three layers are depicted: (1) Perception layer, where physical devices reside; (2) the translation between Internet of Things (IoT) protocols and Hyper-Text Transfer Protocol/Secure (HTTP/S) is made at the Accessibility layer and; (3) the Virtual Object layer is comprised of virtual representations of physical devices, each Virtual Object (VO) embodies a template that describes the behaviour for physical devices. Straight lines represent direct connections between nodes, which means that no intermediate protocol or data exchange format is needed (nodes at the Accessibility layer are responsible for performing such actions). Dashed lines represent that the VO at one end of the line handles the physical device at the other end of the line. Finally, black straight lines represent that nodes are connected through HTTP/S and that there exists an HTTP/S Application Programming Interface (API) compatible with nodes at both ends of the line. Green, straight lines represent that nodes are connected through IoT protocols, such as Bluetooth Low Energy (BLE).

The objective of this paper to provide an abstraction mechanism to describe and execute the behaviour of Things in any WoT architecture. This section explains the architecture used in our proposal. There is a common trend on IoT architectures to represent physical devices with virtual representations or agents that enhance their capabilities. Such virtual representations are called Virtual Object (VO) [16]. Virtual Objects (VOs) are usually placed in fog or cloud middleware [16,17,18]. The behaviour templates developed in this work are interpreted and executed where those VO reside, thus more resources are available to execute the behaviour that the templates describe. The first layer in the seminal work of the WoT [2] defines an Accessibility Layer that enables each physical device to the web by translating each IoT protocol to HTTP/S and providing a common RESTful (REpresentational State Transfer) Application Programming Interface (API) to interact with them. As seen in Figure 1, we assume that physical devices are exposed via HTTP/S in order to interact with the VOs. A basic division between layers can be made. The first layer is composed of physical devices, which is the Perception layer. The second layer is responsible for enabling physical devices to the web, the Accessibility Layer. The third layer, the Virtual Object layer, is composed of VOs that interact with the Perception layer via the Accessibility layer. As explained in [2] and in the W3C WoT Architecture [5], Things can become Web Things (web-enabled Things) by three mechanisms. If they have enough computing resources to implement the HTTP/S protocol, they can be directly connected to the web. Otherwise, they can use Gateways (GWs) or cloud/fog infrastructures that translate their proprietary protocols or non-standard data exchange formats into web standards. The template developed in this paper is embodied by a VO. Each VO is given the capability to interact with physical devices through the Accessibility layer. Therefore, actions encoded by an HTTP/S call the Accessibility layer, which in turn informs the physical device to actuate in the real world. Thanks to this action abstraction, relationships can be made between one VO and multiple physical devices. It is only necessary to change the Uniform Resource Identifier (URI) actions refer to; this is explained in more detail in Section 5.3. The architecture is shown in Figure 1 where different settings can be appreciated. The following lists the settings, from left to right:

1. The first setting is composed of two smart light bulbs from different brands (they use different communication protocols and/or data exchange formats) and a light switch at the Perception layer. There is a Raspberry Pi at the Accessibility layer that translates IoT protocols from the physical devices to an HTTP/S API. Finally, three VOs dwell in the VO layer and each one embodies a template. Note that the same template is used for each one of the VOs that communicate with the smart light bulbs, which means that the same template is used to control both of them. The template for the light switch describes the behaviour for controlling (turn on/off) both smart light bulbs. The control is made at the VO layer. More precisely, the switch VO sends commands and receives messages from the two VOs that represent the smart light bulbs.
2. The second setting is composed of a smartphone at the Perception layer that directly connects to a VO at the VO layer through HTTP/S. To lighten the image, the template the VO embodies is not drawn. The interaction is made possible via an app installed at the smartphone.
3. The third setting comprehends a thermostat and an air conditioner machine at the Perception layer. They both connect to an Arduino board at the Accessibility layer, which exposes their functionality as an HTTP/S API. In this scenario, a single VO is responsible for managing the thermostat and the air conditioner. This can be achieved by simply using both Uniform Resource Locators (URLs), *https://.../thermostat* and *https://.../airc*, as the target for the template actions. Finally, this VO communicates with the VO that represents the smartphone, enabling for controlling the temperature of the room.

The model architecture depicted in Figure 1 shows the interaction patterns between physical devices and our proposal (templates) in a WoT architecture. One of the features of interest in our proposal is that it is middleware-agnostic. Therefore, critical challenges such as full-fledged security and privacy are implemented by concrete middleware. For example, the VICINITY project [19] defines a Virtual Neighbourhood and is a platform that links various ecosystems providing interoperability as a service for infrastructures in the IoT. It tackles privacy by allowing the owner of Things to define sharing access rules of devices and services. The communication between VICINITY Nodes occurs between a Peer-to-Peer (P2P) network based on those rules. This network supports VICINITY Nodes with security services such as end-to-end encryption and data integrity [20]. The IoT ecosystems are connected to VICINITY through the VICINITY Node. Hence, security and privacy outside VICINITY is handled by the manager of the IoT ecosystem. Security in that ecosystem could be an exchange of identifiers and encryption keys between VOs and physical devices or gateways, representing the weakest phase when security could be compromised.

Integration with other middleware involves three main efforts: (1) to implement an engine to interpret the flow of execution defined by the FSM template (which can be reused by middleware developed with the same programming language); (2) to adapt the input and output channels of the FSM engine with an HTTP/S interface provided by the middleware; and to provide an RDF and SPARQL API to the FSM engine.

**Figure 2:**
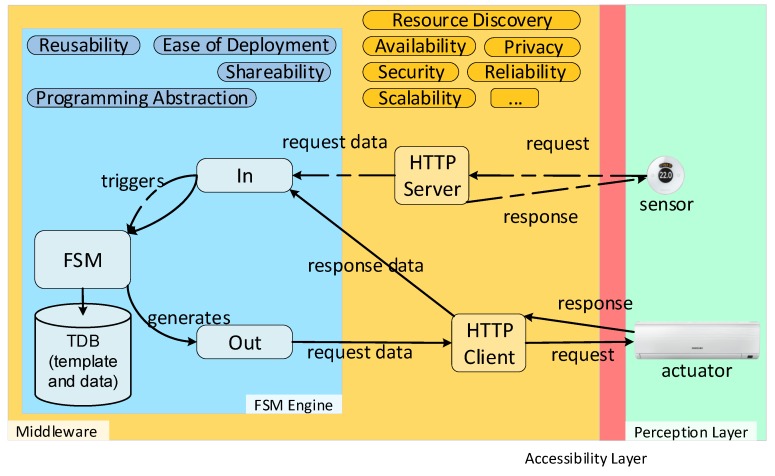
Functional blocks for third-party middleware integration. The layers are: Finite-State Machine (FSM) Engine and Third-party middleware (Middleware), Accessibility Layer and Perception Layer. The FSM Engine complements the middleware. Only two components are mandatory in the middleware layer to enable our approach. Arrows indicate the flow of information between components in each layer. Dashed lines represent the sensor pathway and straight lines represent the actuator pathway.

As shown in Figure 2, our approach complements third-party middleware by providing several desired properties. By combining RDF and the model of execution flow that FSMs provide, we enable a programming abstraction. The programming abstraction can be shared among multiple engine implementations in different middleware. The ease of deployment is facilitated by the fact that behaviour and executor are decoupled. Behaviours for web-enabled physical devices can be created ad hoc when needed and deployed, as the necessary software to execute the behaviour already exists. A Triplestore Database (TDB) or RDF API and SPARQL endpoint is also needed for the FSM engine in order to store the template and save the data received during runtime. Guards, conditions and actions use these data (see Section 5). As seen in Figure 2, our approach relies on HTTP/S in order to enable communication with the Perception Layer. Therefore, it is mandatory for third-party middleware to have an HTTP/S interface. Two pathways are shown in Figure 2. The first pathway assumes a sensor (thermostat/thermometer) with an HTTP/S client that pushes data to the middleware. The HTTP/S server in the middleware receives this request and sends it to the input interface of the FSM engine and triggers the FSM logic, which is read from the template instance. The FSM does not return any response with data as it may not be available at that time. However, the middleware is still able to respond to the HTTP/S request. The second pathway is the actuator pathway. There, the actuator is directly addressable as it is an HTTP/S server. Following the process triggered by the sensor, the FSM may generate an action to turn on the actuator. For example, if the temperature is higher than 27 ºC, the course of action would be to turn on the air conditioner. This logic and action are written in the FSM template instance. The FSM generates an action which is sent to the HTTP/S client provided by the middleware. An HTTP/S request is sent to the actuator and the actuator may respond with valuable data, which is then used as the input of the FSM.

## 4. An Example to Model

**Figure 3:**
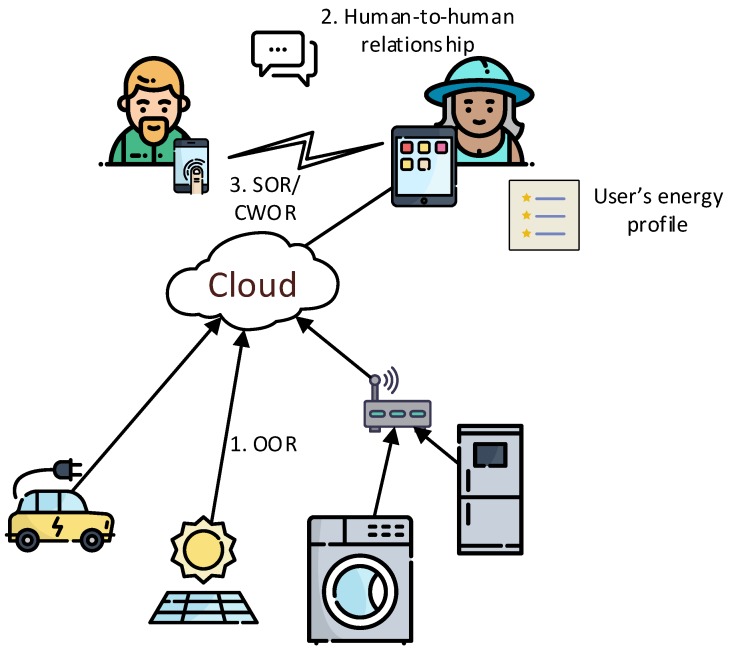
Prosumer Community Group formation leveraging Social Internet of Things. Firstly, prosumers and appliances establish an Ownership Object Relationship (OOR) via the augmented Social Virtual Object (SVO) (1). When prosumers come close together and establish and interact (2), their devices establish Social Object Relationship (SOR) or Co-work Object Relationship (CWOR) relationships and exchange the energy profiles of the prosumers (3).

This section introduces the use case that is modelled throughout the paper. Our objective is to model a template for a VO that allows users to exchange their energy profile information and check if they are compatible. For the sake of simplicity, we define “compatibility” of energy profiles as “similarity” of energy profiles. The use case is framed in the context of the Social Internet of Things (SIoT), which endows devices with the ability to belong to social networks of devices. The Lysis [21] platform is an implementation that enables the SIoT. There, VOs are extended to become social and create relationships; they become Social Virtual Objects (SVOs). Several types of relationships can be created. We are interested in Ownership Object Relationship (OOR) and Social Object Relationship (SOR) relationships. OORs are established among heterogeneous objects that belong to the same user. SORs are defined when objects come into contact (close range) because their owners come in touch with each other during their lives. The overall context of the use case we want to model is as follows:

1. A user interested in being part of a prosumer community to benefit from the community and achieve certain goals installs a discovery app on his/her smartphone. The smartphone becomes an SVO [21].
2. The potential Prosumer Community Group (PCG) user installs apps or allocates apps in the cloud that enable retrieving data from his/her energy-related devices (devices that produce or consume energy), enabling social capabilities for each one (SVO).
3. Devices and Distributed Energy Resources (DERs) that consume and/or produce energy owned by an owner establish an OOR relationship (Figure 3, 1). This relationship is then leveraged to obtain energy-related data from DERs that belong to the same owner. These data can be both aggregated and stored or stored individually for each device.
4. The data obtained is used to build an energy user profile during a time period. The profile is then stored as part of the discovery app, and thus enabling the smartphone and other social devices to retrieve such information. A social device is a device that the user (prosumer) usually carries with himself/herself.
5. When the user establishes a social relationship with another potential user (Figure 3, 2), both social devices (i.e., smartphones) also establish social relationships (Figure 3, 3) and exchange energy profiles and goals. Then, the SVOs determine if they are compatible or not.

Our objective is to model the behaviour expressed in Item 5. The model starts describing the steps that take place when one SVO meets another SVO and ends when the compatibility results between them have been exchanged. The initial peering process is assumed to be done by a service implemented in Lysis [21]. In addition, the energy profile is supposed to exist in the device. The process is described as:

1. Wait for a peer to be available.
2. Send the energy profile to the peer.
3. Wait until the peer’s energy profile is received.
4. Check the compatibility between the device’s and peer’s profiles.
5. Inform of the compatibility result to the peer. The results are exchanged because the compatibility check of each device could be different.
6. Wait until the peer’s compatibility result has been received.

**Figure 4:**
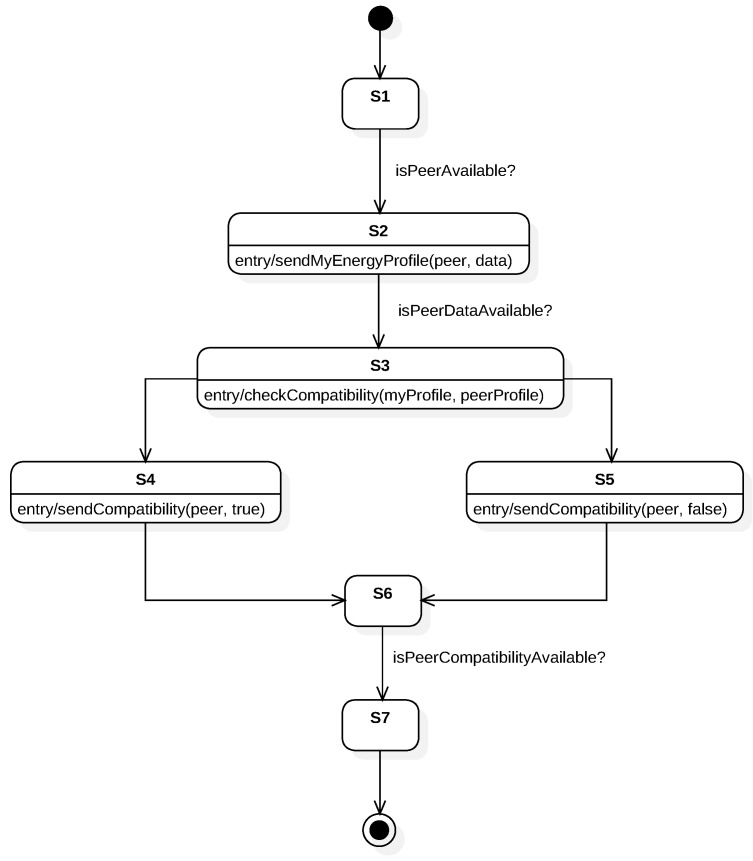
Finite-State Machine representation using Unified Modeling Language.

To model the behaviour, we have chosen an FSM because it enables us to describe a sequence of actions to be executed and the use of conditions to modify the execution’s flow. In order to visualize the FSM, we first model the behaviour using StarUML [22] following UML standards. Figure 4 shows the execution flow using states and conditions. The aim of UML is to provide a standard manner to model the design and behaviour of a system; it is made to be comprehended by humans and not by machines. However, our interest is to enable machines to interpret and execute this behaviour. OWL provides explicit meaning, making it easier for machines to automatically process and integrate information. From the options discussed in Section 2, we have chosen the FSM ontology developed in [13]. Some properties and classes have been added to integrate it with the web, others have been renamed to follow W3C naming conventions on ontologies. The modified ontology is used to describe the system’s behaviour. Note that the FSM is designed in a manner that both VOs (each one interacting with one of the physical devices shown in Figure 3) share the same behaviour.

## 5. Behaviour Modelling Using Ontologies

Throughout the rest of the paper, the prefix fsm: is used to shorten its URI. The siot: prefix represents some classes and properties specific to the domain of the use case. Finally, the: prefix is used to reference the file or ontology where the own model is stored.

### 5.1. Skeleton of the FSM

The first step is to define the skeleton of the FSM, it contains the instance of the machine, its states and its transitions.

As shown in Listing 1, an FSM is defined by the class fsm:StateMachine and the states as fsm:State. The initial state is also defined as fsm:InitialState, the final state as fsm:FinalState and intermediate states as fsm:SimpleState, these three classes extend from fsm:State. An fsm:StateMachine instance should have their states associated with the property fsm:hasStateMachineElement. Each state can have some exit or entry actions; they are associated with the property fsm:hasEntryAction or fsm:hasExitAction.

The transitions between states are described by the class fsm:Transition. As seen in Listing 2, a transition has a source state with the property fsm:hasSourceState and a target state with fsm:hasTargetState. A transition can also contain none or more guards associated with the property fsm:hasGuard.

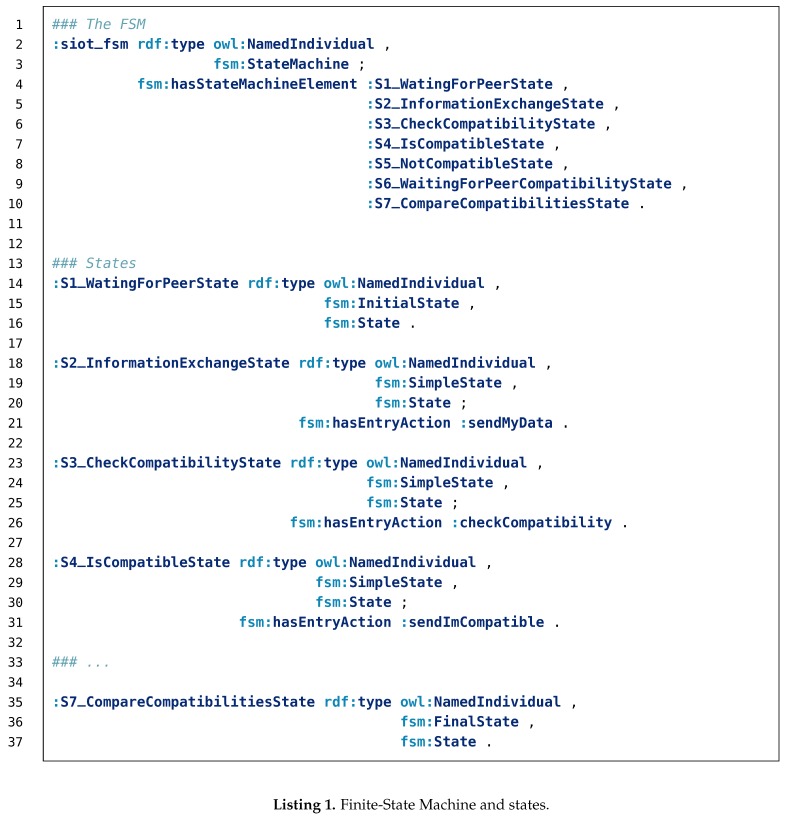



### 5.2. Guards and Conditions

The purpose of a guard, defined by the class fsm:Guard, is to provide a method to evaluate if the transition has to be travelled. The guard can contain none or more conditions to express what has to be evaluated. It can also have none or more actions to be executed if the guard evaluates true.

A condition is defined with the class fsm:Condition; it contains a body object marked as fsm:Body that represents the condition’s content. This body has a string associated with fsm:hasContent that contains the actual condition represented as a SPARQL query. The body should also include its type associated with fsm:hasBodyType to indicate the type of the body’s content. Even though it is possible to use different types, an ASK SPARQL query is needed when used on conditions, as the main focus is to work directly with RDF data and ontologies. The reason to use the type property is that the body object is also used as the body and the value for the header “Content-Type” to be sent on HTTP/S requests, where it can take other formats such as RDF or JavaScript Object Notation (JSON). Some conditions are modelled on Listing 4. The procedure to evaluate the transitions is:

- Retrieve all the state’s exit transitions. Note that only one of these transitions should be true at the same time, otherwise different executions of the same FSM may lead to different results if the order of the evaluation of transitions changes.
- For each transition, retrieve its guards (Listing 3). If it has no guards, then that transition is considered feasible. If any guard is true, then the transition is also feasible, which means that an OR condition is applied between all the guards. The utility of having multiple guards is to have different actions that are executed only under certain cases and not each time the transition is travelled.
- For each guard, retrieve its conditions. Another OR operation is applied between these conditions, and if any of them is true, the guard also evaluates to true. It is important to evaluate all the guards and to not stop when one of them is true, as all the guards’ actions must be executed if their guard is true.
- The body of each condition should be an ASK query. An ASK query is a type of query that contains a pattern of triples, it searches for a set of triples that match the pattern. If any matching set is found, the query returns true, and it is false otherwise. The query is evaluated against the RDF database. The condition evaluates the same as the ASK query.

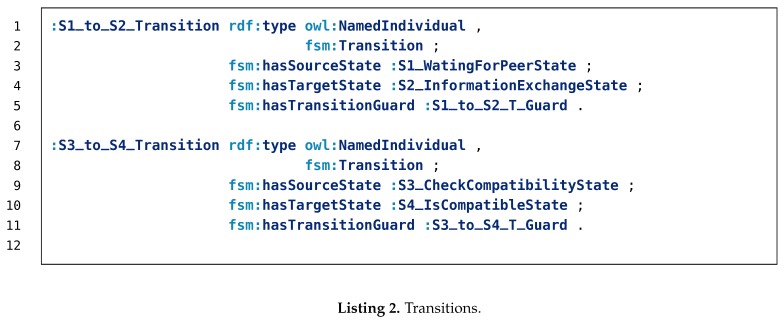

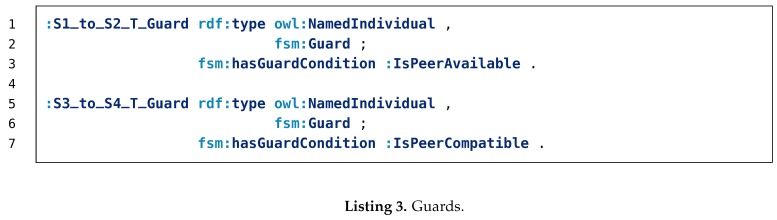

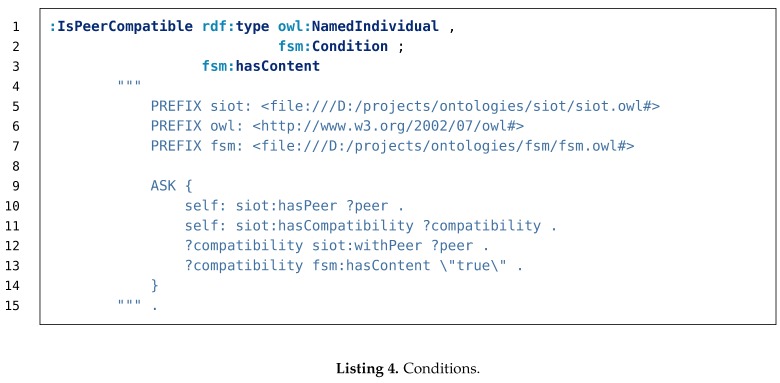

### 5.3. Actions

An action requests or sends data via HTTP/S to another node in the architecture. The HTTP Vocabulary in RDF 1.0 [23] ontology by W3C is used to define actions. An action is defined with the class fsm:Action and http:Request at the same time. An action has a body, associated with fsm:hasBody, which is sent with the request. The body follows the same structure used in the conditions; it can have different types like RDF (Listing 5), JSON and others. Moreover, a timeout can be specified to limit the amount of time that an action waits for the response, it is specified in milliseconds with the property fsm:hasTimeoutInMs. A request method like GET or POST is indicated with the property http:mthd. This property references an object and not a literal, W3C has already defined the standard method objects in [23], these objects are the preferred ones to use. Therefore, as templates are not bound to perform actions using the same URI, a template can perform actions on multiple nodes. This, in conjunction with the FSM model, enables the templates or, more precisely, their instantiation, the VOs, to interact with multiple physical devices and services, such as other VOs. This is the case of the third scenario described in Figure 1, where a single VO is able to orchestrate two different physical devices. The VO also communicates with the smartphone described in the second scenario via its VO.

**Figure 5:**
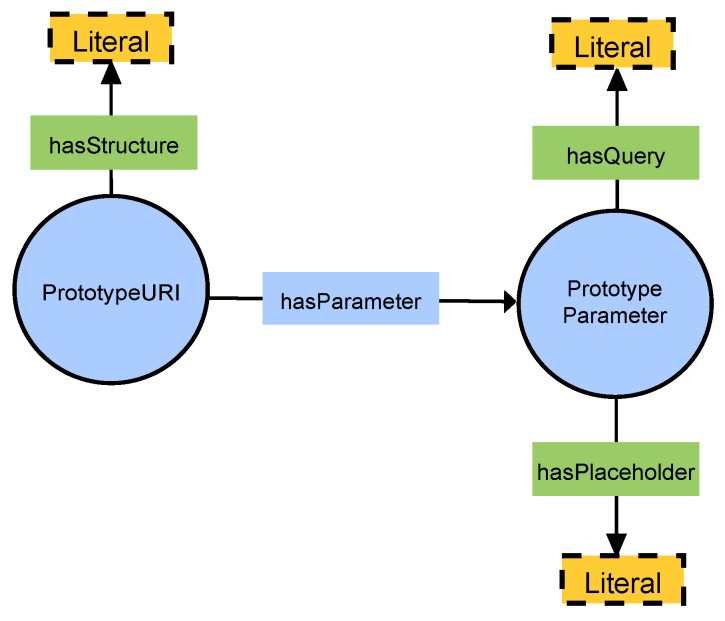
Schema of PrototypeURI.

The action has a URI to express where the request is done. This URI can be expressed in different ways; our two approaches are:

- Use an absolute URI with the property *http:absoluteURI* to express the final address as in Listing 6. This is used when we know in advance where the request is done and that the address will not change.
- Use a prototype URI with the property fsm:hasPrototypeURI, shown in Listing 7. A prototype is an object of the class fsm:Prototype that defines the structure of a URI with one or more placeholders that are replaced during runtime. A schema of the classes and properties involved is presented in Figure 5. This is used when we do not know in advance the final address or when it changes frequently. An absolute URI is computed from the prototype each time the action needs to be executed. The replacements of parameters are not done on the ontology; this newly computed URI is built separately in program memory. A prototype defines the URI’s structure with a string linked with fsm:hasStructure, for example *http://localhost:9000/[fsm_id]/send_data*, where *[fsm_id]* is the placeholder. The prototype has an fsm:Parameter for each placeholder, linked via the property fsm:hasParameter. The purpose of the parameter is to indicate what placeholder needs to be replaced and to provide a function that generates the value to fill the placeholder. Each parameter has the placeholder itself as a string (like *[fsm_id]*) linked with fsm:hasPlaceholder and a SPARQL query as a string linked with fsm:hasQuery. The query must be a SELECT query that returns only one value. The value returned by the query is used to replace on the URI the placeholder with the same parameter key.

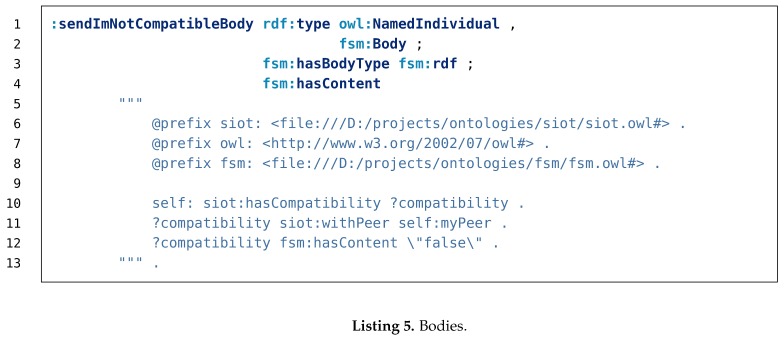

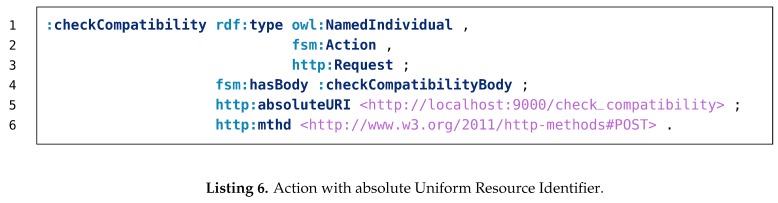

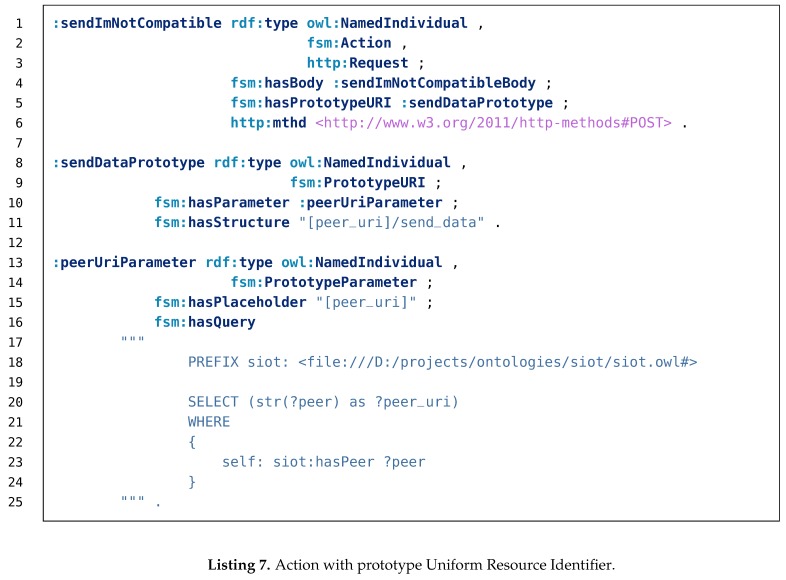

## 6. Execution

This section explains how the proposed ontology-defined behaviours can be executed. We have implemented the template interpreter and embedded it in a web server. We have used Java as the programming language to implement the template interpreter and executor. Apache Jena [24] has been used as the TDB and SPARQL engine. Play Framework 2 [25] has been used to implement the web service. Akka [26], an Actor Model toolset, has facilitated the task of deploying the two VOs used in the example and enabled us to endow the engine with a reactive programming model. This means that the engine only “wakes up” when there is a new request for that VO, that is when new data is available. Both Play Framework 2 and Akka ease the task of implementing asynchronous actions. As explained in Section 5.3, the amount of time an action waits for a response (HTTP/S response) can be specified. Nevertheless, actions are performed sequentially and asynchronously. If actions $A_{1}$ and $A_{2}$ with fsm:hasTimeoutInMs 1 s and 5 s respectively are to be performed when entering a state, both actions will execute at the same time, without $A_{2}$ waiting for $A_{1}$ response. No other FSM operation like checking guards will be performed until the actions finish. An action finishes if it receives a response within its response timeout or it has not received any response during that time. We have made this implementation decision to prevent the VO from being blocked while waiting for the response.

**Figure 6:**
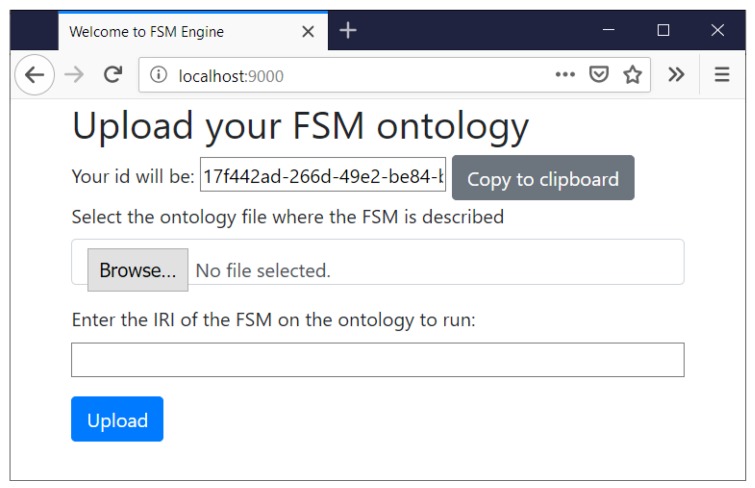
Graphic interface where users can upload their models of Finite-State Machines. Users are requested to load their Finite-State Machine (FSM) ontology and the Internationalized Resource Identifier (IRI) that identifies the concrete instance of the FSM. Users are also provided with an ID that uniquely identifies the Virtual Object (VO) that will use the FSM.

As shown in Figure 6, users are provided with a graphic interface where they can upload their models. Users are asked to upload the file with the FSM ontology and the Internationalized Resource Identifier (IRI) that identifies the instance of fsm:StateMachine to be executed. When accessing the web the user is given an ID that identifies the FSM instance. This ID is also used to identify the VO’s unique endpoint that it will be listening HTTP/S requests from. The VO is also able to send HTTP/S requests to both web interfaces of physical Things and other VOs. Each VO is provided with a RDF database that stores all the data received and generated during the execution of the FSM. The RDF database uses a base prefix “:” that uniquely identifies the data. The IRI of this prefix is built from the server URL (e.g., *http://localhost:9000/*) and the previous auto-generated ID. The IRI has a # appended at the end to refer to objects inside this domain. The final IRI looks like *http://localhost:9000/[fsm_id]#*.

In order to ease how conditions and actions are built, the VO automatically adds the RDF database’s “:” prefix to all RDF data and SPARQL queries that are written on the ontology or sent to the server. The VO also adds a prefix called “self:” that refers to the same IRI that “:” but without the final #, so the FSM instance is able to reference itself. The IRI of self: is then *http://localhost:9000/[fsm_id]*.

The endpoint of the VO provides an API with three generic actions that enable communication with the FSM instance:

- Get data: it returns the result of a SPARQL SELECT query. The query is sent to the server on the request’s body. It is a GET request and the URI is *http://localhost:9000/[fsm_id]/get_data*.
- Send data: it saves RDF data on the RDF database. The RDF data to be stored is sent on the request’s body. It is a POST request and the URI is *http://localhost:9000/[fsm_id]/send_data*.
- Execute operation: it executes a SPARQL query like INSERT on the RDF database. The query to execute is sent on the request’s body. It is a POST request and the URI is *http://localhost:9000/[fsm_id]/execute_operation*.

After the user sends the data via the initial form, a VO is deployed. During the deployment process, the FSM ontology is read and pre-loaded. The process is as follows:

1. Load the FSM ontology into Apache Jena.
2. Search on the model and read the FSM instance of type fsm:StateMachine that has the IRI specified by the user.
3. Read all the states and states’ actions. All entry and exit actions are also read.
4. For each state, read the transitions that have the state as the source are searched. For each transition, retrieve their guards, conditions and guards’ actions.

In the initialization process, the VO applies default values to some properties if they are not specified on the ontology:

- Action timeout: if no timeout is specified for an action, the VO sets a default timeout of 1 second for that action.
- Action method: if no method is specified for an action, the VO sets the method to GET.
- Action body: if the body is not specified, it is set to an empty string.
- Body type: if no type is specified for a body, it is treated as plain text.

After the initialization process, the VO starts to execute the FSM. The model explained in Section 4 and modelled through the paper has been executed using the prototype implementation. Two VOs are deployed and instantiated using the same template. Conditions *isPeerAvailable?*, *isPeerDataAvailable?* and *isPeerCompatibilityAvailable?* are fulfilled by external services. Those services are mocked as the main purpose of the prototype implementation is to validate the feasibility and consistency of our approach. They return true or the necessary data so the FSM can transition to the next state. In order to visualize the execution flow of the FSM in real time via a web browser, we have endowed the VO with a WebSocket interface.

## 7. Use Case Analysis

This section analyses several use cases to assess the suitability and to identify the strengths and weaknesses of our solution.

### 7.1. Use Cases

We have considered seven use cases with different qualitative characteristics. Those use cases are gathered or related to European projects where the authors participate. Three of them relate to the Healthcare IoT for equipment tracking, personal monitoring and medicine dispense, they are related to the Advanced Training in Health Innovation Knowledge Alliance (ATHIKA) project. One of them considers logistics in Industry 4.0, gathered from the project Strategic Programme for Innovation and Technology Transfer (SPRINT). The aim of both projects is to transfer knowledge, and thus a learning enhancement system is analyzed. The Smart City use case involving intelligent trash bins is related to the ENVISERA project. Finally, an analysis is performed over the use case explained in Section 4 which is related to the Smart Grid [17,27], it is referred to as the “Prosumers” use case.

We also provide a qualitative analysis of the effort needed to integrate our approach in different types of middleware. In order to do that, we classify middleware in use cases according to the types described in [28,29]. We assume that they support the HTTP/S protocol both as server and client. Healthcare middleware is considered service-based. Service-based middleware is more secure than cloud-based middleware in that cloud security and privacy are managed by the cloud provider. However, end-to-end encryption is still challenging in IoT middleware. Actor-based middleware provide the capability of embedding lightweight actors in resource-constrained, thus enabling security capabilities to end devices. Actor-based middleware is not considered for healthcare use cases as many challenges are still to be solved [28]. An event-based middleware is considered for the use cases of logistics in Industry 4.0, intelligent trash bins and prosumers. Concretely, an event-based and actor-based (no embedded actors in the physical device) middleware is assumed [17]. Event-based architectures are recurrent in Smart City, Industry 4.0 and Smart Grid systems as they allow massive data ingestion. Finally, the prototype implementation presented in Section 6 is considered to be used between web-enabled devices and a Learning Management System (LMS) system.

#### 7.1.1. Hospital Equipment Tracking

Hospital equipment, like stretchers and wheelchairs, can be tracked to obtain its position and whether they are being used or not. The tracking is used to optimize the utilization of resources, to keep track of the inventory and to reserve the equipment. Our VO can be used to represent each piece of equipment. The VO will be asking the sensors for the current position and for other values like the weight they are carrying. For instance, when the VO detects a significant change on the weight it can send a message to a centralized service.

#### 7.1.2. Hospital Personal Monitoring

On a hospital, patients identity, status or position can be monitored through IoT to increase their safety. The medical staff can also be tracked to optimize their workflow. The VO used for the patient and the staff are different and there is a one-to-one relationship between any user (patient or staff) and a VO. The VO can be used to retrieve the current position and patients’ vital signs. If the patient exits their room or some value of a vital sign gets out of a healthy range, the VO can send a notification to the medical team. The staff position is also useful, for example, to alert the professional closer to the patient.

#### 7.1.3. Intelligent Medication Dispenser

An intelligent medication dispenser is a machine that gives patients the correct medication at the right time and also checks for incompatibilities between medicines. This is a perfect tool to avoid problems like the ones explained by the authors in [30] such as a lethal combination of medicines, recalled medicines, not taking medicines on the right time or taking the same dose twice. The VO can implement the logic of deciding which medicines to dispense. First, it will wait for the identification of the patient (for instance, with an Radio-Frequency IDentification (RFID) card). Second, the VO will retrieve the information about the medication assigned to the patient by a doctor (this information has been previously added). Then, it will select the medication that needs to be taken at that time and verify that it had not been taken already. The VO also checks the compatibility with other medicines. It uses an external database that describes medicine compatibility in RDF format. Then, the VO executes a SPARQL query with the necessary reasoning logic. If any incompatibility is found, the patient is notified. The VO can be also programmed to alert patients at the time they have to take the medication through a smart-alarm or a smartphone.

#### 7.1.4. Logistics on Industry 4.0

Logistics on warehouses can be made more efficient through automatic processing of the incoming items. The author in [31] introduces a use case where all pallets of items have an RFID tag. The RFID tags are read on their arrival by RFID readers. Multiple tags can be read at the same time as their position on the pallet is not important as long as they are in the range of the reader. The tag can provide information like the product identifier, the number of items in the pallet or additional information like fragile content or notes written by the sender or the driver. The stock of the warehouse is updated through an Enterprise Resource Planning (ERP) application. A VO can implement this process. For each RFID tag processed, the VO sends the information of the tag to the ERP system. The VO can ask the ERP for the best place to store the incoming item or it can even implement the logic of deciding the best place to store it via queries and reasoning. Finally, the VO will notify a worker or robot to carry the item to its storage place.

#### 7.1.5. Learning Enhancement

The learning performance of students can be improved by monitoring the environment of the classroom and the status of the student. The work done in [32] explains a way to achieve this; they use the technology of Wireless Sensor Networks (WSNs) to integrate temperature, humidity, illumination, the concentration of CO_2_ sensors and human emotions detection cameras on wireless networks. Some parameters like temperature are used to automatically adjust the room temperature, but others like the students’ mood may need the attention of the professor. A VO can be used to actively monitor all the parameters and adjust the conditions of the room to optimal values; it can also alert the professor about the students that are losing focus. We assume that the VO has a one-to-one relationship with the classroom (temperature, humidity, CO${}_{2}$, ...) and one-to-many with the students in a classroom, but it is a relationship with a reduced and limited set of entities (students).

#### 7.1.6. Intelligent Trash Bin

The intelligent trash bin is an already used technology on Smart Cities that monitors the state of trash bins and enables optimization on the waste collection. Authors in [33] propose an architecture that implements this technology; the bin contains an Arduino and attached sensors that collect information about waste level, temperature, humidity, motion and weight. The information of the sensors is sent to the cloud where patterns to optimize the collection of waste are extracted. A previous step can be achieved using edge computing to analyze the physical state of the bin to prevent heavy damage, as the analysis on the cloud may not be real time. The one-to-one or one-to-many relation between a VO and an intelligent trash bin or set of trash bins (if they are placed close to one another) can be established. The VO is placed on the edge cloud. Each time, the VO receives the report from the Arduino, before sending it to the cloud, it will analyze the values of humidity, toxicity (with additional sensors), motion or weight to detect potential problems like fire, vandalism/bad use or a possible toxic hazard. If any of these problems are detected, the VO sends an alert to the trash management system.

### 7.2. Analysis and Discussion

This section provides a qualitative analysis of the described use cases. A summary of the analysis is provided in Table 1. The following metrics are considered to provide the analysis:

- Time reliability. This metric considers the limitations of the technologies that have been used to develop our solution, namely RDF, SPARQL and HTTP/S. They allow interoperability and shareability, the main focus of our proposal. However, they may be computationally heavy, especially SPARQL. This solution may not be suitable for time-critical applications. Specialized systems are better suitable to run time-critical applications. The speed of the network due to congestion can be mitigated with a dedicated Local Area Network (LAN). A Low score means that the VO cannot keep up with the timing requirements of the use case. A High score means that the use case does not need accurate timing and that some delay is acceptable.
- Scalability. Scalability represents how the VO performance scales with the number of Things that it manages. The total performance of the system degrades when the number of Things controlled increases. Usually, the performance degradation is linear and the biggest impact will be at time reliability because the available resources will be shared between more Things. Having more Things to control usually means that there will be more states and actions to perform, and that will translate in more delays. A Low score means that the manner in which the system is designed provokes a performance degradation as more Things are integrated into the system.
- Reusability. We consider reusability as the degree of how easy it is to reuse the generated template or templates for other similar use cases. For example, a template used by a VO that senses the room temperature and actuates accordingly to increase the comfort of the inhabitants can be easily used on other rooms or buildings. It may be only necessary to change the temperature thresholds. The temperature is represented as the value to be queried and not hardcoded in the execution flow or template. This example has a High reusability. On the other hand, a template that describes the behaviour of a robot that assembles car pieces has Low reusability. There will be substantial differences in the assembly logic of different models of cars and between companies.
- Suitability. Suitability is the degree of fitness between the behaviour design that the current FSM ontology allows and the desired functionalities/behaviour of the use case. If none or few modifications are needed, the suitability is considered High. If major modifications are needed, then suitability is Low.
- Incompatibilities (with domain-specific ontologies). The degree of incompatibilities between the base ontologies used by the proposed solution and the ontologies of the use case domain. A score of High means that it is hard to integrate our solution with domain-specific ontologies. It is possible to have a score of None if there are no incompatibilities.
- Outsourcing. Outsourcing refers to the need to use external services that provide operations that the FSM cannot do. If the VO is independent or uses few external services, then the outsourcing is considered Low. VOs that act as service aggregators have high outsourcing. The value of this metric does not have an explicit positive or negative meaning, its purpose is to describe a characteristic of the VO behaviour.
- Query complexity. Query complexity is an estimation of how heavy the queries of the use case are. Heavy means that the number of returned triples is very high or/and the query execution time is expected to be long (more than half a second). A High complexity means that heavyweight queries will be performed during the execution flow.
- Knowledge complexity. Knowledge complexity refers to how much knowledge about ontologies, SPARQL and behaviour representation with FSMs the user requires to implement the system for a concrete use case. A High score means that the user necessitates a High degree of knowledge to represent the behaviour of the system. A Low score denotes that few experiences are needed as it is easy to represent the behaviour.
- Integration Effort. Integration effort considers the required effort to integrate our system in the middleware/architectures assumed for each use case. If the middleware supports actors or VOs, the effort is considered Low or Medium.

Time reliability is High in the cases where it is not important if the system has delays greater than one second, for instance, it is not critical if the VO of the medicine dispenser has a delay of two seconds. It is Medium where a second of difference is important like monitoring the health of a patient (a fast alert could save the patient’s life). In the case of Logistics, it also critical if, for each item to be unloaded, there is a delay of some seconds because a lot of items are expected to be processed fast and that can sum up to a big delay.

Scalability gets a Medium score on hospital personal monitoring because adding more Things to the VO may cause bigger delays that are not desired. This is because if more constants are to be monitored on the patient, the tasks and logic that the VO performs increase.

Reusability is Medium on the logistics use case as the desired behaviour about how the items are stored and processed may change for each company or warehouse. The idea behind templates is to define a behaviour abstracted from the physical implementation to provide reusability. It is also Medium in the use case of hospital personal monitoring as each hospital may have different workflows to optimize the performance of the staff. Reusability is High in other use cases as we consider that the behaviour/execution flow can be defined generically.

Suitability is High in all use cases as the VO is able to execute the desired behaviour. There are no incompatibilities between our proposed solution and domain-specific ontologies. Our solution relies on ontologies in order to be as compatible as possible with other ontologies. Domain-specific ontologies are expected to extend higher level ontologies such as SOSA/SSN in order to promote template reusability.

Outsourcing is Medium where the system has some operations that rely on external processes. The medication dispenser relies on an external catalogue in order to check medicine compatibility. The logistics use case scores Medium at outsourcing because it relies on the ERP to perform its tasks; some operations are too complex to be implemented by a FSM or need specialized software.

Finally, query complexity is Low in almost all cases as the queries are usually with local data and are simple. It is Medium on the medication dispenser because there may be some heavy queries that check the compatibility between all the medications of the patient. Knowledge complexity is Medium on the dispenser as the queries for medicine compatibility check can be complex to write. On the logistics, the queries can be also complex if the ERP does not provide the location to store the items; queries of searching the optimal place should be written in this case.

Our approach is easily applicable in learning enhancement, intelligent trash bin and prosumers use cases. We consider that they have a generic structure (High reusability) and that the modelling of their behaviour is straightforward with Low query and knowledge complexity. They are not time-critical use cases so they score High in time reliability. Their score is High on scalability, as each VO has a relation one-to-one or one to a reduced and limited set of entities to control. Prosumers use case has a Medium score on Outsourcing as it uses external services to get informed if a peer is available or for the complex operation of checking the energy profile compatibility.

The integration effort with any concrete middleware comprehends (1) implementing the FSM engine in the programming language used by the middleware; (2) implementing a software adapter to connect the FSM inputs and outputs to the HTTP/S server and client interfaces of the middleware; and (3) providing an RDF and SPARQL API to the FSM engine. If the middleware provides a TDB, it is only necessary to connect the FSM engine to the database. Otherwise, it needs to be implemented. For that reason, the integration effort is considered Medium in almost all use cases and respective type of middleware. Once this initial effort is done, the effort remains to create the template of the desired behaviour using the ontologies and considerations described in this manuscript. Regarding the learning enhancement use case, a simple web service like the one presented in Section 6 can be used as the orchestrator between web-enabled devices and the LMS. Note that the purpose of the prototype is only to provide a web execution environment to the FSM engine. It lacks common web security and privacy mechanisms such as authentication and authorization. If the integration between our approach and the middleware is not possible or requires too much effort (e.g., in middleware that does not have the concept of VO/(Web-)Thing), our approach can be used at the application layer as a microservice that composes with the middleware’s HTTP/S API.

## 8. Conclusions and Future Work

Given the heterogeneity that characterizes the IoT, a novel middleware-agnostic approach that allows for describing and executing the behaviour of devices has been proposed and implemented. The objective is to allow the reusability and shareability of the execution flow among multiple and heterogeneous IoT deployments. The approach relies heavily on existing standards to promote interoperability and reusability. FSMs are used as the model to create the execution flow using ontologies. Our work is contextualized in a reference architecture recommended by W3C, the W3C WoT Architecture. We have used the concept of VO, which are virtual counterparts of physical devices as the computational entities that run the concrete instances of the templates. Several use cases have been analyzed to asses the viability and suitability of our solution. Relying on standards such as RDF, SPARQL and HTTP/S has some drawbacks. They tend to be more heavyweight than an ad hoc solution. For example, HTTP/S can be replaced by Constrained Application Protocol (CoAP) (a lightweight, IoT version of HTTP/S) and the data model can be replaced by a SQL or NoSQL database. For that reason, our approach is not well suited for time-critical applications such as monitoring and reasoning over patients’ vital signs in a hospital. However, our approach is well-suited for IoT scenarios that are non-time-critical and with a low level of variability between each deployment. It allows for reusing behaviours for heterogeneous physical devices with the same set or subset of capabilities.

In future works, we expect to fully apply a TD interface to the VOs. Our aim is to extract and generate part of the TD definition from the FSM instance. This will also enable the alignment with other ontologies such as the oneM2M Base Ontology and enable service composition. Given that each state has its own inputs and outputs, research is needed to identify which Properties, Actions and Events (according to the TD model) should be exposed. SPARQL performance is computationally heavy and our solution only allows to send SPARQL queries to external services. We plan to add the capability of sending non-SPARQL requests to external services described using ontologies such as TD ontology or oneM2M Base Ontology. Our goal was to facilitate deployments in similar IoT scenarios. Nevertheless, the task of creating the template ontology can be tedious, especially if the template has multiple states and actions with prototype URIs. Our approach has some restrictions imposed by the FSM model and the WoT architecture (we assume that physical devices expose an HTTP/S API). Therefore, we plan to create a visual tool to hasten the creation of FSM templates using visual building blocks like in StarUML. The IDE will be used to create the template in a visual manner and to automatically translate the visual representation to the ontological one.

## Figures and Tables

**Table 1:** Use cases benchmark.

	Hospital Equipment Tracking	Hospital Personal Monitoring	Intelligent Medication Dispenser	Logistics on Industry	Learning Enhancement	Intelligent Trash Bin	Prosumers
Time reliability	High	Medium	High	Medium	High	High	High
Scalability	High	Medium	High	High	High	High	High
Reusability	High	Medium	High	Medium	High	High	High
Suitability	High	High	High	High	High	High	High
Incompatibilities	None	None	None	None	None	None	None
Outsourcing	Low	Low	Medium	Medium	Low	Low	Medium
Query complexity	Low	Low	Medium	Low	Low	Low	Low
Knowledge complexity	Low	Low	Medium	Low/Med.	Low	Low	Low
Integration Effort	Medium	Medium	Medium	Medium	Low	Medium	Medium
